# Look but don't touch: Visual cues to surface structure drive somatosensory cortex

**DOI:** 10.1016/j.neuroimage.2015.12.054

**Published:** 2016-03

**Authors:** Hua-Chun Sun, Andrew E. Welchman, Dorita H.F. Chang, Massimiliano Di Luca

**Affiliations:** a School of Psychology, University of Birmingham, Birmingham B15 2TT, UK; b Department of Psychology, University of Cambridge, Cambridge CB2 3EB, UK; c Department of Psychology, University of Hong Kong, Hong Kong

**Keywords:** Roughness, Glossiness, Visual material, fMRI, MVPA

## Abstract

When planning interactions with nearby objects, our brain uses visual information to estimate shape, material composition, and surface structure *before* we come into contact with them. Here we analyse brain activations elicited by different types of visual appearance, measuring fMRI responses to objects that are glossy, matte, rough, or textured. In addition to activation in visual areas, we found that fMRI responses are evoked in the secondary somatosensory area (S2) when looking at glossy and rough surfaces. This activity could be reliably discriminated on the basis of tactile-related visual properties (gloss, rough, and matte), but importantly, other visual properties (i.e., coloured texture) did not substantially change fMRI activity. The activity could not be solely due to tactile imagination, as asking explicitly to imagine such surface properties did not lead to the same results. These findings suggest that visual cues to an object's surface properties evoke activity in neural circuits associated with tactile stimulation. This activation may reflect the a-priori probability of the physics of the interaction (i.e., the expectation of upcoming friction) that can be used to plan finger placement and grasp force.

## Highlights

• Secondary somatosensory area responds to tactile-related visual properties.
• Visual inputs are necessary to elicit this somatosensory activation.
• This visual-somatosensory crossmodal network may facilitate action planning.

## Introduction

When we look at objects, we are able to predict how they will feel once we come into contact with them. For instance, shiny objects with *glossy* surfaces, like silverware and plastic, are expected to feel smooth and hard when pressed, and sliding our fingers over their surface may generate stick–slip interactions. Textured objects, like a tree bark and sandpaper, are expected to feel rough when pressed upon and can lead to abrasion if stroked. Matte objects, like wood and stone, are expected to feel irregular and can generate skin vibration if caressed. These expectations refine movement planning, e.g., slippery objects necessitate a more precise and powerful grip.

While these issues have been appreciated at the conceptual and theoretical levels (Fleming, 2014, Xiao et al., 2013), work examining the neural underpinnings of visual appearance has mainly concentrated on responses from classically defined visual responsive cortical areas. Human brain imaging work and electrophysiological recordings have suggested the importance of ventral cortical areas in processing information about surface textures and material categories (Cant et al., 2009, Cant and Goodale, 2007, Cavina-Pratesi et al., 2010a, Cavina-Pratesi et al., 2010b, Goda et al., 2014, Hiramatsu et al., 2011). However, given the potential importance of visual cues in driving the nature of our interactions with nearby objects, the role of somatosensory areas during visual surface perception is still unclear. Previous studies have shown that the somatosensory cortex is important for tactile perception of texture and roughness (Kaas et al., 2013, Kitada et al., 2005, Pruett et al., 2000, Roland et al., 1998, Sathian et al., 2011, Simões-Franklin et al., 2011, Stilla and Sathian, 2008). Here we ask whether this region responds also to visually presented information about similar surface properties.

Several groups have identified areas in human visual cortex whose activity relates to tactile and haptic stimuli. In one human fMRI study, object-sensitive regions in occipitotemporal cortex (including the lateral occipital region (LO) and posterior fusiform sulcus (pFs)) were identified to represent information about object weight when lifting visually presented objects. Moreover, after learning that object textures were associated to object weight, this texture–weight association was also represented in occipitotemporal areas (Gallivan et al., 2014). A second fMRI study has similarly shown haptic object-selective activity in occipitotemporal cortex (Amedi et al., 2001). Further studies have found haptic texture-selective responses in the middle occipital cortex and haptic shape- and location-selective responses in intraparietal sulcus (IPS) (Sathian et al., 2011, Stilla and Sathian, 2008). These results suggest that occipitotemporal areas, middle occipital cortex, and IPS are actually not strictly visual, but bimodal as they are capable of representing haptic information as well. Thus, it is possible that crossmodal activations may exist for other primary sensory areas, i.e. visual texture-selective responses may also be found in somatosensory cortex.

To test whether somatosensory areas respond to visually defined textures, we measured human fMRI responses to visual images of computer-generated objects that had perceptually different surface characteristics. The stimuli were designed to evoke a visual impression of surface gloss or roughness, while the control conditions were designed to depict stimuli with similar image statistics that nevertheless gave rise to a different impression of surface properties. All the stimuli were novel objects to avoid issues of remembered sensations. We used multivoxel pattern analysis (MVPA) to test for visual and somatosensory areas that contained neuronal responses that supported reliable discrimination of different visual surface characteristics. Our rationale was that if the brain has a system to generate expectations of tactile sensations when looking at objects with distinctive surface properties, changes in appearances that affect such expectations should elicit different activation responses in somatosensory cortex. Indeed, we found this to be the case. In a control experiment, we further show that imagining such surface properties is alone insufficient to generate similar somatosensory activations.

## Materials and methods

### Participants

Sixteen participants who had normal or adjusted-to-normal vision were recruited for the experiments. One was the author H.-C. S. and the remaining participants were naive to the tasks and purpose of the study. All were screened for visual acuity and MRI safety before being invited to participate. The age range was 18–39 years old, and 5 of the 16 participants were male. All participants gave written informed consent before taking part in the experiment. The study was conducted according to the protocol approved by the STEM Ethical Review Committee of the University of Birmingham. After completing the experiment, all participants (except the author) received monetary compensation or credits.

### Apparatus and stimuli

#### Stimuli

**Fig. 1:**
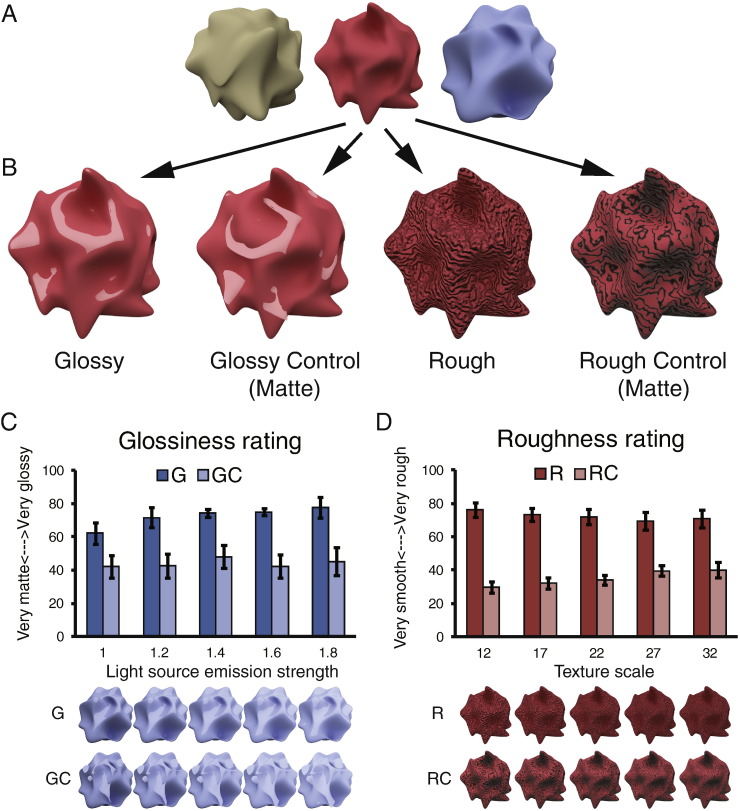
(A) The three objects used to create the stimuli shown to participants. (B) The four experimental conditions: Glossy, Glossy Control, Rough, and Rough Control rendered on one example object. Specular components were shown in Glossy condition (90% diffuse and 10% glossy components) while the highlight areas were rotated and moved in the Control condition to break the impression of surface gloss. In the Rough condition, wave textures were applied on objects' 3-D geometry; in the Rough Control condition, the same wave textures were applied to the reflectance of the surface to create the impression of a smooth surface with a painted texture. Glossiness rating and rough rating results are presented, respectively, in (C) and (D) under five levels of the emission strength from the light source and five levels of wave texture scale. The bars reflect mean rating scores across 7 participants with ± 1 SEM.

The study comprised three 3-D shaped objects generated by Blender 2.67a selected from a previous study (Sun et al., 2015) (Fig. 1A). Stimuli were 12 deg. in diameter on average, and they were presented on a mid-gray background. We created versions of the stimuli for each object that made up the four conditions of the experiment: Glossy, Glossy Control, Rough, and Rough Control (Fig. 1B). In the Glossy condition, objects were rendered using a mixed shader with 90% diffuse and 10% glossy components. In Glossy Control condition, the specular components rendered on Glossy objects were rotated by 45 degs in the image plane, which made the objects look matte since the important contextual information for gloss perception had been destroyed (Anderson and Kim, 2009, Kim et al., 2011, Marlow et al., 2011). In the Rough condition, wave textures were applied to objects' 3-D geometry, resulting in bumps on the surface. In the Rough Control condition, the same wave textures were applied to the objects' surface colour, resulting in a painted texture. In Glossy and Glossy Control conditions, there were five levels of the emission strength from the light source: 1, 1.2, 1.4, 1.6, and 1.8 (Fig. 1C). In Rough and Rough Control conditions, there were five levels of wave texture scale: 12, 17, 22, 27, and 32 (Fig. 1D). The five levels of each object were presented in a random order to reduce adaptation of the fMRI response. A black fixation dot (dia = 0.5 deg) was shown during fixation blocks.

In the control experiment, 12 new objects were presented to participants in familiarisation session before entering the scanner. The 12 objects were split in 4 groups that were rendered with a clear colour–condition association (i.e. blue objects were Gloss, red objects were Rough Control, etc). Then, participants were presented with only the contours of the previously seen objects that were filled with homogeneous colour. Participants were asked to imagine the surface properties of the four conditions specified by the colour. The colour-coding of Glossy/Glossy Control and Rough/Rough Control was counterbalanced across participants. Participants were trained to associate the colour cues with the four conditions and were able to make colour–condition associations with 100% accuracy prior to entering the scanner (and upon re-test after the scan). During the scan, there were five levels of luminance scale for each object contour presented in a random order to reduce any adaption effect in the fMRI response, as in the main experiment.

#### Apparatus

The same apparatus were used as described in our previous paper (Sun et al., 2015). Psychtoolbox (Brainard, 1997) was used for stimulus presentation. A JVC DILA SX21 projector was used for projecting stimuli on a translucent screen inside the bore of the magnet. Participants viewed stimuli via a mirror fixed on the head coil with a viewing distance of 64 cm. Luminance outputs were linearised and equated for the RGB channels separately with colorimeter measurements. A five-button optic fibre button box was used to collect participants' responses in the 1-back task.

#### MRI data acquisition

A 3-Tesla Philips scanner and an 8-channel phase-array head coil were used to obtain all MRI images at the Birmingham University Imaging Center (BUIC). T1-weighted high-resolution anatomical scans (175 slices, TR 8.4 ms, TE 3.8 ms, flip angle 8 deg., voxel size: 1 mm^3^) were obtained for each participant. Functional whole brain scans with echo-planar imaging (EPI) sequence (32 slices, TR 2000 ms, TE 35 ms, voxel size 2.5 × 2.5 × 3 mm, flip angle 80 deg., matrix size 96 × 94) were also obtained for each participant. The EPI images were acquired in an ascending interleaved order for all participants.

### Design and procedure

#### Subjective rating task

Seven naive participants were recruited for the rating experiment. Participants performed glossiness ratings on all Glossy and Glossy Control stimuli in one block and roughness rating on all the Rough and Rough Control stimuli in another block. The order of the two blocks was balanced across participants. Participants viewed stimuli presented on a CRT monitor with a viewing distance of 83 cm. Luminance outputs were linearised and equated for the RGB channels separately with colorimeter measurements. The diameter of the stimuli was 12 deg. Each image was presented for 500 ms after which participants were given unlimited time to rate the image along a scale of “very glossy” to “very matte” for glossiness rating block, or along a scale of “very rough” to “very smooth” in the roughness rating block. Participants were permitted to place their rating bar in any position between the two ends to indicate their rating and the rating value was calculated by computing the distance between the bar and one end divided by the whole scale length.

#### fMRI session

**Fig. S1:**
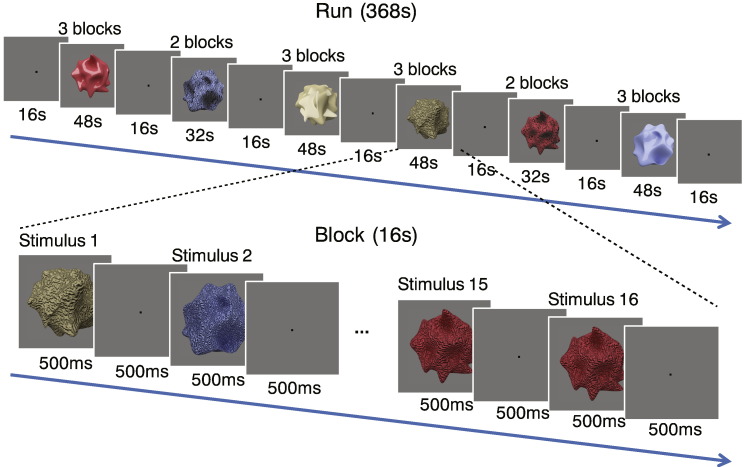
The procedure of one scan run. In each run, there were 23 blocks (16 s each), including 7 fixation blocks and 16 experimental blocks. During each experimental block, stimuli were presented for 500 ms with interstimulus interval (ISI) 500 ms. Participants were instructed to perform a 1-back matching task during the scan by pressing a button.

A block design was used. Each participant took part in 9 or 10 runs with 368 s length of each run in a 1.5-h session. Each run started with four dummy scans to prevent start-up magnetisation transients and consisted of 16 experimental blocks each lasting 16 s. There were 4 block types (i.e., one for each condition), repeated four times in a run. During each block, fifteen objects were presented once in a pseudo-random order and one of them was shown twice (the “event” to which participants had to respond). Stimuli were presented for 500 ms with 500 ms interstimulus interval (ISI). Participants were instructed to maintain fixation and perform a 1-back matching task, whereby they pressed a button if the same image was presented twice in a row. They were able to perform this task well (mean d′ = 2.07; SEM = 0.10). Five 16 s fixation blocks were interposed after the third, fifth, eighth, eleventh, and thirteenth stimulus blocks to measure fMRI signal baseline. In addition, 16 s fixation blocks were interposed at the beginning and at the end of the scan, making a total of seven fixation blocks during one experimental run. An illustration of the scan procedure is provided in Fig. S1.

The same block design was used in the control experiment, except that before the start of each block, a colour dot (the same colour as the object contours present next) was presented for 4 s for cuing the surface property that should be imagined. Participants were also instructed to perform a 1-back matching task while imaging surface property. They were able to perform this task as well (mean d′ = 2.48; SEM = 0.16) as in the main experiment.

### Data analysis

#### Functional MRI data processing

The basic data processing procedures for both the main and control experiments were the same as in our previous study (Sun et al., 2015). We also computed global signal variances of blood oxygenation level dependent (BOLD) signal as before and removed the scan runs, which exceeded 0.23% of global signal variances. Seventeen runs out of 153 runs across 16 participants in the main experiment and 2 runs of 60 runs across 6 participants in the control experiment were excluded from further analysis based on this criterion.

#### ROI definition

Regions of interest (ROIs) were defined using separate localiser scans. For early retinotopic visual cortex (areas V1, V2, V3, V3A, V4), we used standard retinotopic mapping based on rotating wedge stimuli and expanding/contracting concentric rings (Abdollahi et al., 2014, DeYoe et al., 1996, Sereno et al., 1995). Area V3B/KO (Dupont et al., 1997, Zeki et al., 2003) was defined retinotopically as the region of cortex with a full hemifield representation located inferior to, and sharing a foveal representation with, V3A (Tyler et al., 2005). This retinopically defined area overlapped with the set of contiguous voxels that responded significantly more (*p* < 10^− 4^) to kinetic boundaries than transparent motion of a field of black and white dots (Dupont et al., 1997). Other groups have identified this region of cortex as area LO1 based on retinotopic mapping techniques (Larsson et al., 2010); however, individual variability in the clarity with which these regions could be identified retinotopically led us to use the V3B/KO designation. The lateral occipital complex (LOC) was defined as the set of voxels in lateral occipitotemporal cortex that responded significantly (*p* < 10^− 4^) more strongly to intact than scrambled images of objects (Kourtzi and Kanwisher, 2000). LOC subregions (LO, extending into the posterior inferotemporal sulcus; posterior fusiform sulcus (pFs), posterior to mid-fusiform gyrus) were defined based on the overlap of functional activations and anatomical structures, consistent with previous studies (Grill-Spector et al., 2000).

**Fig. 2:**
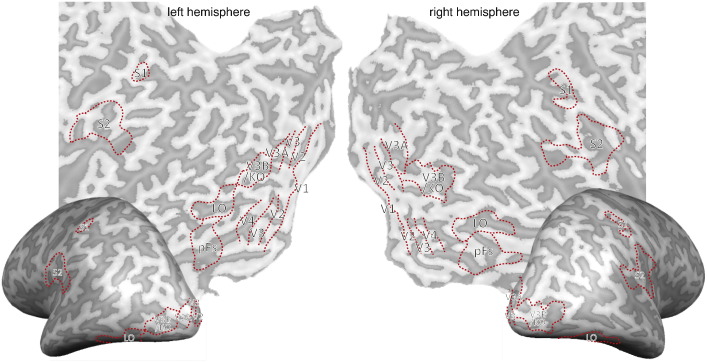
Red dotted lines are the ROI boundaries we defined with independent localisers for a representative participant for visual cortex. For somatosensory cortex, red dotted lines show the boundaries of group activation with *p* < 10^− 4^ threshold under air-on versus air-off contrast.

Somatosensory areas were defined by a somatosensory localiser adapted from a previous study (Huang and Sereno, 2007). This separate localiser session consisted of 20 blocks, 10 air-on blocks and 10 air-off blocks showed alternately, each lasting 16 s. In the air-on blocks, air puffs were delivered at the participants' ten fingertips through plastic tubes (6 mm inner diameter) from below a board in cycles (1 s on 1 s off). No air was delivered in air-off blocks. Somatosensory areas were defined by contrasting activations in air-on blocks with air-off blocks. Primary somatosensory area (S1) was defined as the more dorsal portion of the activations around Brodmann area 1-3b and the secondary somatosensory area (S2) was defined with as the more ventral portion of the activations around the parietal opercular areas OP1–OP4. The coordinates of Brodmann areas 1-3b and OP1 were acquired from the SPM Anatomy toolbox (Eickhoff et al., 2005, Eickhoff et al., 2006, Eickhoff et al., 2007a, Geyer et al., 1999, Geyer et al., 2000). The centres of these areas were converted from MNI space in SPM to Talairach space in BrainVoyager. We present detailed coordinate information in Table 1. The mean S1 centre is consistent with the centres of Brodmann area 1-3b (Geyer et al., 1999, Geyer et al., 2000) and the mean S2 centre is consistent with the centre of OP1 (Eickhoff et al., 2006, Eickhoff et al., 2007b). All the ROIs were defined by the independent localisers shown in Fig. 2.

#### fMRI analysis

We used multivoxel pattern analysis (MVPA) to compute classification accuracies for different experimental conditions. For voxel selection, all voxels in each visual area were arranged with *t* value larger than 0 for the contrast of “all experiment conditions *vs.* fixation block” voxels in GLM *t*-value maps. Voxels of somatosensory areas were defined by significant *t* values for the contrast of “air-on *vs.* air-off” voxels in GLM *t*-value maps. The top 250 voxels were selected for classifications across ROIs. If a participant had fewer than 250 voxels in one ROI, we used the maximum number of voxels that had *t* values greater than 0. After selecting the voxels, their time series was extracted and converted to z-scores. Then, the voxel-by-voxel signal magnitudes for a stimulus condition were obtained by averaging the signals over 8 time points (TRs) (= 1 block) separately for each scanning run. Before averaging, the time series was shifted 4 s to account for the hemodynamic response delay. The global baseline differences of these response patterns across the stimulus conditions and scanning runs were excluded by subtracting the mean of the patterns. These block-averaged signals were used as response pattern in an ROI for the classification analysis. We used a linear support vector machine (SVM) to discriminate between activities evoked by the different conditions in each ROI. In the training phase, 32 response patterns for each of the stimulus conditions were used as a training dataset for those participants that completed 9 runs and 36 response patterns were used for those who completed 10 runs. Then, 4 response patterns for each condition were classified by the trained classifier in the test phase. These training/test sessions were repeated and validated by a leave-one-run-out cross-validation procedure. The prediction accuracies were defined as the average of these cross-validation classifications. The mean accuracies across participants were then tested against shuffled baseline with Bonferroni corrected, one-tailed single-sample *t*-test, to check whether they are significantly above chance level (0.5 for all classifications in this paper as they are all binary classifications). Shuffled baselines were calculated with permutation tests (1000 repetitions for each ROI of each participant with randomly shuffling stimulus condition labels per test. The one-tailed, upper 95th percentile boundaries of accuracy distributions were averaged across all ROIs).

## Results

**Fig. S2:**
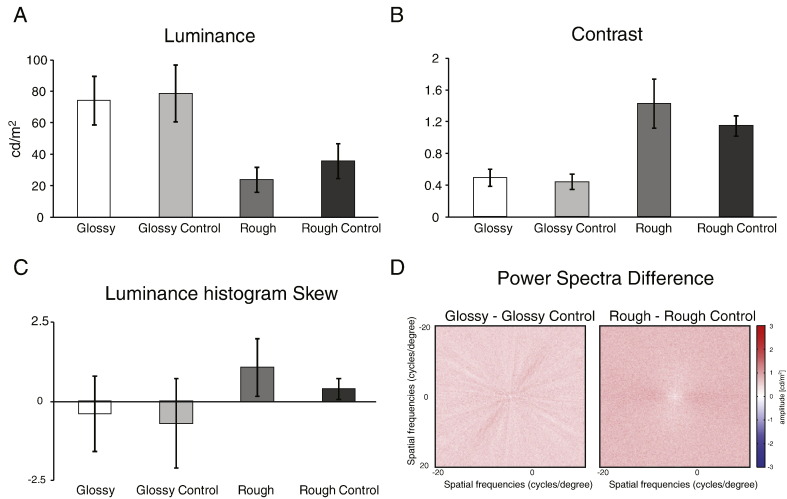
Image statistics of (A) pixelwise luminance, (B) contrast, (C) histogram skew of the four conditions. Luminance was calculated by averaging the mean luminance of all pixels in each image then averaging across images. Contrast was calculated with pixelwise luminance's standard deviation divided by its mean for each image, averaged across images. Skew was calculated as the third standardised momentum of the luminance histogram of each image, averaged across images (Motoyoshi et al., 2007). (D) Difference in power spectra across the 15 images (3 objects × 5 levels) calculated for each image pair and then averaged across images.

We presented participants with novel irregular objects (Fig. 1A) that were rendered to depict different surface characteristics in the four different experimental conditions: Glossy, Glossy Control, Rough, Rough Control (Fig. 1B). To ensure that participants experienced different impressions of surface gloss or roughness in the different conditions, we first performed a psychophysical experiment asking participants to rate either the glossiness (Fig. 1C) or the roughness (Fig. 1D) of the presented objects. We found that the control versions of the stimuli were effective in reducing the appearance of gloss/roughness (two-tailed Wilcoxon signed-rank test between Gloss and Gloss Control: *Z* = − 2.2, *p* < .05; between Rough and Rough Control: *Z* = − 2.4, *p* < .05). Moreover, to ensure that—despite different impression of surface properties—Glossy and Rough objects had similar image statistics of pixelwise luminance, contrast, histogram skew, and power spectra, we quantified image statistics across conditions, finding that the control versions of the stimuli were well matched to their counterparts (Fig. S2).

**Fig. 3:**
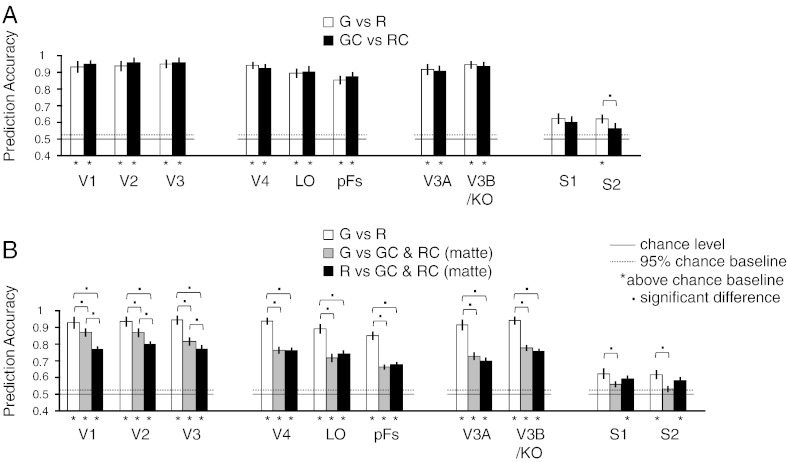
Classification performance of MVPA across 16 participants for (A) Glossy *vs.* Rough (white bars) and Glossy Control *vs.* Rough Control (black bars); (B) Glossy *vs.* Rough (white bars), Glossy *vs.* Matte (gray bars), and Rough *vs.* Matte (black bars). The bars reflect mean classification accuracy with ± 1 SEM. Chance level was 0.5 for all classifications as shown in solid horizontal lines. Dotted horizontal lines represent the upper 95th percentile with permutation tests (see “Data analysis”). The one-tailed, 95% boundaries of accuracy distributions were averaged across all ROIs, which were 52.31% for G *vs.* R, 52.37% for GC *vs.* RC, 51.95% for G *vs.* M, 53.80% for R *vs.* M). Asterisks below the bars represent significant above-chance accuracies (single-sample *t*-test, one-tailed, Bonferroni corrected, *p* < .05). Dots above the bars show significant difference between the contrasts with Tukey's HSD post-hoc tests (*p* < .05).

To test for brain areas that might respond differentially to surface characteristics depicted in the different conditions, we used a block-design protocol to measure fMRI responses in independently localised regions of interest in retinotopic visual cortex (V1, V2, V3, V4, V3A), object-related areas (LOC), an area linked with the processing of gloss (V3B/KO), and somatosensory cortex (S1, S2). To analyse the data, we used multivoxel pattern analysis (MVPA) to discriminate fMRI responses evoked by the different conditions. We found that we could reliably decode differences between Glossy *vs.* Rough stimuli in most of the regions of interest we had localised (Fig. 3A, white bars above the permuted chance baseline). Contrasting the Glossy Control and Rough Control conditions allowed reliable predictions to be made across the visual cortex, although performance in somatosensory cortex was not reliably above chance (Fig. 3A, black bars). We ran a 2 (G *vs.* R and GC *vs.* RC) × 10 (ROIs) repeated-measures (r.m.) ANOVA to compare the difference between the two contrasts. We found a significant ROI effect (*F*_9,135_ = 91.5, *p* < .001) and a significant interaction between the two factors (*F*_9,135_ = 2.1, *p* = .031). Tukey's HSD post-hoc tests (*p* < .05) showed that only in area S2 the decoding performance was significantly higher for the Gloss *vs.* Rough comparison than for the Glossy Control *vs.* Rough Control comparison. This suggests that in area S2, the difference between glossy and rough surfaces that is evoked by the visual displays is greater than between control conditions that are similarly smooth.

In addition, we tested for differences in the ability of our classification algorithm to decode surface gloss and roughness. In particular, we used MVPA to contrast Glossy *vs.* Rough conditions as before, as well as the Glossy condition and the Rough condition against Matte conditions (corresponding to the combined Glossy Control and Rough Control conditions). We could reliably decode differences between Rough *vs.* Matte and Rough *vs.* Glossy stimuli in most of the areas we had localised (Fig. 3B, black bars and white bars above the permuted chance baseline). Glossy *vs.* Matte also showed a similar pattern in visual areas; however, performance in somatosensory cortex was not reliably above chance (Fig. 3B, white bars). To compare the difference between the three contrasts, we ran a 3 (G *vs.* R, G *vs.* M and R *vs.* M) × 10 (ROIs) r.m. ANOVA. We found a significant difference between the three contrasts (*F*_2,30_ = 45.9, *p* < .001), a significant ROI effect (*F*_9,135_ = 70.3, *p* < .001), and a significant interaction between the two factors (*F*_18,270_ = 9.0, *p* < .001). Tukey's HSD post-hoc tests (*p* < .05) revealed that performance in discriminating Glossy *vs.* Rough conditions was higher than in Glossy *vs.* Matte and in Rough *vs.* Matte for all the visual areas, while for somatosensory areas, Glossy *vs.* Rough was only higher than Glossy *vs.* Matte but not higher than Rough *vs.* Matte. In sum, the pattern of prediction accuracies differed in somatosensory areas from that in visual cortex. First, the performance in discriminating Glossy vs. Matte conditions was at chance. Second, the discrimination of Rough *vs.* Matte conditions was reliable and not significantly different from discriminating Glossy *vs.* Rough conditions. These results indicate that this somatosensory area processes visual surface information at the mesoscale level rather than microscale level (Ho et al., 2008), as Rough *vs.* Matte and Glossy *vs.* Rough are different in the former while Glossy *vs.* Matte is different in the latter.

These data suggest that differential activation of area S2 can be driven by visually presented information. However, we should also consider an alternative possibility that the route to activity in S2 might be somewhat indirect. In particular, it is possible that viewing the stimuli simply caused the participants to imagine the surface of the objects, with this tactile imagery responsible for the fMRI responses we recorded. We therefore conducted an additional experiment in which we instructed participants to imagine objects with different surface construction, to assess fMRI responses in our regions of interest. Prior to conducting the scan, participants were trained (to 100% accuracy) to associate images with one of the four surface characteristics (Gloss, Matte, Rough, or Textured). In particular, participants viewed the contours of the objects that were presented in the main experiment. These shapes were filled with a homogenous colour that was paired with the surface property that participants should imagine.

**Fig. 4:**
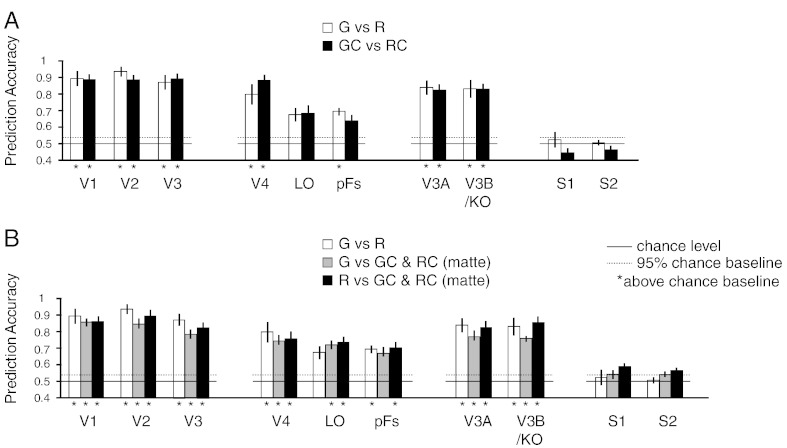
Classification performance of MVPA in the control experiment for the same contrasts as in Fig. 2. Dotted horizontal lines represent the upper 95th percentile with Permutation tests (one-tailed, 95% boundaries of accuracy distributions were 53.80% for G *vs.* R, 53.83% for GC *vs.* RC, 53.15% for G *vs.* M, 53.13% for R *vs.* M). Asterisks in the bottom of the bars represent significant above-chance accuracies (one-tailed Bonferroni corrected single-sample *t*-test, *p* < .05).

We found that decoding performance of differences in the visual appearance of the object was possible across visual regions of interest (Fig. 4)—this was expected as both the colour and shape of the visually presented objects differed across conditions. By contrast, performance in somatosensory areas dropped to chance (Fig. 4), indicating that imagining the different surface properties per se did not support reliable decoding of fMRI responses. In addition, to compare the difference between the two contrasts in Fig. 4A and the three contrasts in Fig. 4B, we ran a 2 (G *vs.* R and GC *vs.* RC) × 10 (ROIs) r.m. ANOVA and a 3 (G *vs.* R, G *vs.* M and R *vs.* M) × 10 (ROIs) r.m. ANOVA, respectively. We only found a significant ROI effect in the 2 (G *vs.* R and GC *vs.* RC) × 10 (ROIs) ANOVA (*F*_9,45_ = 44.2, *p* < .001) and in the 3 (G *vs.* R, G *vs.* M and R *vs.* M) × 10 (ROIs) ANOVA (*F*_9,45_ = 26.3, *p* < .001). Importantly, we did not observe significant differences across contrasts and interactions in both cases (*p* = .216 and p = .052). The marginal interaction effect of the 3 × 10 ANOVA might be due to different performance between visual areas and somatosensory areas across the three contrasts. That is, visual areas had better performance for Gloss *vs.* Rough than for Gloss *vs.* Matte in general while somatosensory areas had nearly chance performance for the two contrasts. Together these results suggest that imagery *per se* is rather unlikely to underlie the responses we measured in area S2. Rather, it seems that viewing objects with different surface properties causes activity in somatosensory cortex with little effort on behalf of the participants.

## Discussion

We tested how surface properties of viewed objects evoke activity in different parts of the cerebral cortex. We found that visually responsive areas of the brain discriminated between different classes of objects, as might be expected. Surprisingly, we found that these same images lead to differential responses in somatosensory cortex: rough and smooth surfaces lead to different patterns of activation in areas that were localised based on their processing of tactile stimuli. These findings suggest that surface properties retrieved from visually presented stimuli activate a visual–somatosensory crossmodal network. We speculate that this network may provide a way to decide whether haptic exploration should take place (Klatzky et al., 1993) and to predict the outcome of our interactions with objects (i.e. predict the friction and weight of the object) so as to facilitate action planning (e.g. determine the required force and precision when picking up nearby objects, (Buckingham et al., 2009).

The differential activations of somatosensory cortex for rough and smooth surfaces shown here is unlikely to be due to confounding cues such as differences in imagery, memory, and other non-tactile visual characteristics. Notably, we showed that rough and smooth surfaces were no longer discriminable by somatosensory cortex if observers were asked to imagine, rather than view, the visual properties (Fig. 4). Visual inputs are therefore necessary to elicit the somatosensory activations seen here and this processing is assumed to be automatic and bottom-up. However, it is still not clear whether the imagery of touching different surfaces (e.g. rough *vs.* glossy) can activate different patterns in S2 or in motor cortex as motor imagery is more involving in action plan and may recruit relevant areas. In addition, the observed effect in somatosensory cortex cannot be associated with remembered sensations as the stimuli we used were novel. Further, the effect cannot be attributed to differences in non-tactile characteristics (e.g. object colour and image features) as although we used different object colours for our four conditions in the control experiment, they did not lead to differential activation patterns in somatosensory cortex. Even when the patterns on the objects were very different (the two control conditions in the main experiment), no difference in somatosensory activation was found. Thus, the activity in somatosensory areas is likely to reflect the specific tactile information (e.g., related to roughness) retrieved from visual cues.

Interestingly, the activation patterns in the somatosensory areas S1 and S2 differed in some respects. Somatosensory responses about visual roughness were particularly pronounced in area S2, where we found significant differences between fMRI responses to Glossy *vs.* Rough and Glossy Control *vs.* Rough Control conditions but there was no significant difference between the two contrasts in area S1. In addition, we could reliably decode differences between Glossy *vs.* Rough stimuli in S2 but not in S1. The differences in response between S1 and S2 are consistent with previous studies that have shown the selective activation of S2 with tactile stimulation (Sathian et al., 2011, Stilla and Sathian, 2008) and greater activation with rougher surfaces in S2 (Pruett et al., 2000, Simões-Franklin et al., 2011).

Our data demonstrate that the representation of surface properties in S2 is not based only on information from one sensory modality, and that its activation does not require tactile input. This finding challenges the view that visual and tactile surface information is processed largely independently as previously inferred by observing the qualitatively different encoding, processing, and representing of texture information in the two modalities (Bergmann Tiest and Kappers, 2007, Eck et al., 2013b, Guest and Spence, 2003, Sathian et al., 2011, Stilla and Sathian, 2008, Whitaker et al., 2008). Instead, our results support the notion of multisensory processing of surface texture and roughness. This implies that surface texture information is represented both in the visual and tactile systems and, in line with evidence from human psychophysics (Baumgartner et al., 2013, Jones and O'Neil, 1985, Lederman and Abbott, 1981, Picard, 2006), to some extent the information can be transferred between the two modalities. Indeed, a recent study showed that content-specific information (e.g. category properties) is retrieved by somatosensory cortex from familiar visual objects that observers had plenty of haptic experience with (Smith and Goodale, 2015). Here we demonstrate that object familiarity is not required; somatosensory cortex responds to visual surface information even if it is novel and cannot be categorised. This suggests that somatosensory cortex may receive information from a visual processing stage that is earlier than that responsible for object organisation and categorisation in higher visual areas such as inferiotemporal cortex. Such a mechanism would allow observers to interact with novel objects or with objects that are difficult to identify or categorise (e.g. partially occluded objects or objects under weak illumination).

Previous visual–tactile crossmodal studies did not identify bi-sensory texture-selective regions in somatosensory cortex (Amedi et al., 2001, Eck et al., 2013a, Sathian et al., 2011, Stilla and Sathian, 2008). Instead, bi-sensory texture-selective areas were found in the middle occipital cortex, left lingual gyrus, left ventral premotor cortex, and left inferior frontal gyrus (Sathian et al., 2011, Stilla and Sathian, 2008). In one human fMRI study, activity in somatosensory cortex was found when stimuli were presented bimodally (vision and haptic), but not when presented unimodally (Eck et al., 2013a). Here we show that somatosensory cortex (specifically S2) can be activated by visual information alone. It is possible that the subtle differences in activation patterns that are captured by the MVPA approach used here could not be captured by previous work that used conventional general liner models or percent signal change analyses (Amedi et al., 2001, Sathian et al., 2011, Stilla and Sathian, 2008). It is still not clear whether visual texture information is represented in the same way as haptic texture information in S2. Future studies can explore this issue by adding tactile counterparts of glossy and rough objects for comparison (e.g. examine whether the response to glossy objects *vs.* rough objects by viewing and by touching involve in the same voxels or not in S2). The visually induced somatosensory activation found here is compatible with an anticipatory system that extracts surface properties from visual information, perhaps in preparation for a possible interaction with it. Such an anticipatory system might be crucial for providing information about surface and material properties that determine friction and dynamic properties (i.e., deformability), which in turn could be considered in planning an action (Di Luca and Ernst, 2014). For example, people expect metal objects to be heavier, stiffer, and smoother than wooden objects and therefore would use more force to contact and grip them (Bergmann Tiest and Kappers, 2014, Buckingham et al., 2009). This assumption can be further examined by testing whether the response in S2 for different visual materials can be modulated by simultaneous tactile inputs (e.g. congruent vs. incongruent tactile inputs). One may argue that no areas related to action planning (e.g. parietal cortex, premotor cortex) were found in the current study; however, we did not ask participants to carry out an action plan, so the absence of these activations is actually expected. We reason that action performance requires two phases: collecting information to form an action plan and subsequently performing the plan. We interpret the S2 activation as belonging to the computations involved in the first phase—collecting relevant tactile information to prepare the action plan in an automatic way. Moreover, previous studies showed that the anticipation of a sensory input activated similar networks as during real sensory stimulation. These networks included S1 (Porro et al., 2002, Porro et al., 2003) and S2 (Carlsson et al., 2000, Porro et al., 2004). These findings suggest that the activation of S2 by visual material cues might implicate the same network that is responsible for tactile perception of surface mesostructure and material.

The mechanism underlying the processing of visual material inputs by S2 might be similar to that activated with observed touching, being touched, or observing other people using tools—interactions that have been proposed to involve the mirror system (Blakemore et al., 2005, Järveläinen et al., 2004, Keysers et al., 2004, Kuehn et al., 2013, Nakano et al., 2012). However, a recent study suggests that the activation related to touch observation found in somatosensory cortex might actually be in posterior parietal cortex (Chan and Baker, 2015), and the locations of S1 and S2 we defined (Fig. 2) are different from superior parietal and inferior parietal regions they defined. Therefore, it is possible that the activation related to visual surface information and the activation related to touch observation involve distinct but closely located regions in somatosensory cortex and posterior parietal cortex, respectively. In our experiment, it is unlikely that participants retrieved tactile information from memory since the objects were all unfamiliar. Rather, we speculate that during their lifetime, humans are exposed to crossmodal associations, i.e., a smooth tactile sensation with shiny objects and high-frequency spatiotemporal stimulation with rough ones. This repeated perceptual stimulation is stored as a sensory association between the tactile sensation and the view of objects' surface—i.e. a coupling prior (Ernst, 2006). This previous work, and the findings we present here, are consistent with the view that not only higher-order association cortex but also early sensory areas which were previously presumed to be unisensory can be modified by multisensory signals (Ghazanfar and Schroeder, 2006, Merabet et al., 2007, Schroeder and Foxe, 2005). Such associations can be reciprocal: for instance, tactile stimulation has been shown to modify activity within the visual cortex of blindfolded participants (Merabet et al., 2007). These findings are also consistent with recent monkey studies which showed that visual areas respond to tactile stimulations alone and S2 (around the upper bank of the lateral sulcus) also responds to visual stimulations alone (Guipponi et al., 2015, Hihara et al., 2015).

In addition to somatosensory cortex, we also found that fMRI responses in certain visual areas supported discrimination between different classes of objects including Glossy *vs.* Rough, Glossy *vs.* Matte, and Rough *vs.* Matte (see Fig. 3). This result is consistent with previous human and monkey neurophysiology evidence about the involvement of early visual areas, ventral visual areas (Georgieva et al., 2008, Köteles et al., 2008, Kovács et al., 2003, Peuskens et al., 2004), and dorsal visual areas (Nelissen et al., 2009) in visual material and texture extraction (Goda et al., 2014, Hiramatsu et al., 2011, Okazawa et al., 2012, Sun et al., 2015, Wada et al., 2014). Note that the differential responses in the three contrasts might also be due to the differences in low-level image features—a factor that we cannot rule out here. Interestingly, classification performance for discriminating Glossy *vs.* Rough conditions was higher than that for discriminating between Glossy *vs.* Matte and Rough *vs.* Matte conditions across all the visual areas, probably because visual differences (from both low-level features and global components) within a class increased after combining the two control conditions (Matte) so that classification performance decreased accordingly. Moreover, earlier areas V1–V3 showed better classification performance for discriminating between Glossy *vs.* Matte than Rough *vs.* Matte conditions, consistent with the evidence for basic image statistics that may act as important cues to surface gloss (Motoyoshi et al., 2007, Okazawa et al., 2012, Wada et al., 2014).

In summary, we found that somatosensory cortex, and in particular area S2, is responsive to the surface characteristics of roughness and glossiness conveyed by visual information. While visual areas respond to both surface properties and low-level image features, we found that area S2 primarily responds to visual surface properties that imply different tactile sensations. This area may constitute part of a circuit that predicts the outcome of our interactions with nearby objects to facilitate action planning.

The following are the supplementary data related to this article.

## Figures and Tables

**Table 1:** Talairach coordinates of S1 and S2 defined by the somatosensory localiser compared with coordinates of Brodmann area 1, 3b, and parietal operculum 1.

		x	y	z
Left hemisphere	S1	− 45.75 ± 3.19	− 23.37 ± 5.41	47.36 ± 6.14
S2	− 50.55 ± 5.14	− 18.31 ± 5.81	17.73 ± 4.49
BA1	− 44.85 ± 10.89	− 28.43 ± 8.76	51.61 ± 10.22
BA3b	− 43.16 ± 9.05	− 23.05 ± 7.76	41.67 ± 9.94
OP1	− 49.66 ± 7.20	− 25.38 ± 3.69	17.77 ± 2.68
Right hemisphere	S1	47.92 ± 4.69	− 22.57 ± 6.84	45.68 ± 6.11
S2	49.90 ± 4.04	− 19.29 ± 5.50	18.39 ± 3.59
BA1	49.5 ± 10.56	− 27.33 ± 9.80	49.52 ± 10.98
BA3b	41.93 ± 13.7	− 25.48 ± 10.63	42.80 ± 13.22
OP1	54.51 ± 7.32	− 23.93 ± 4.24	17.63 ± 2.64

Mean ± SD of x, y, z coordinates of S1 and S2 were calculated across 16 participants. The coordinates of BA1, 3b, and OP1 were obtained through the SPM Anatomy toolbox (Eickhoff et al., 2005, Eickhoff et al., 2006, Eickhoff et al., 2007a, Geyer et al., 1999, Geyer et al., 2000) and transformed to Talairach space.
